# Relationship between ASPECTS-region and clinical outcome in patients with large vessel occlusion stroke: a *post-hoc* analysis of randomized clinical trial

**DOI:** 10.1097/JS9.0000000000003327

**Published:** 2025-09-25

**Authors:** Xin Xiong, Dahong Yang, Junfang Wan, Yan Yang, Shitao Fan, Changwei Guo, Xiang Liu, Jie Yang, Linyu Li, Gaoming Li, Wenjie Zi, Weilin Kong, Fengli Li

**Affiliations:** a Department of Neurology, Chongqing Hospital of Traditional Chinese Medicine, Chongqing, China; b Department of Neurology, Xinqiao Hospital and The Second Affiliated Hospital of Army Medical University, Chongqing, China; c Department of Anesthesiology, Xinqiao Hospital and the Second Affiliated Hospital of Army Medical University, Chongqing, China; d Department of Anesthesiology, Second Affiliated Hospital of Chongqing Medical University, Chongqing, China

**Keywords:** Alberta stroke program early CT score, endovascular thrombectomy, ischemic stroke, outcome, the large vessel occlusion stroke

## Abstract

**Background:**

The relationship between the infarct region and prognosis can assist clinicians in perioperative decision-making. However, the time (early or extended window) of the stroke may also influence the identification of prognostically relevant regions. We aim to determine whether topological information captured by CT ASPECTS contributes prognostic value in different time windows.

**Methods:**

A *post-hoc* analysis of the DEVT and RESCUE BT studies was conducted. Pretreatment ASPECTS was evaluated by Fast-Processing of Ischemic Stroke software. Patients who had infarcts in at least one ASPECTS region (ASPECTS ≤ 9) were included and categorized according to treatment time window (early, 0–6 hours versus extended, 6–24 hours from last known well to puncture). The primary outcome measure was poor clinical prognosis (90-day modified Rankin Scale score of 3–6). Multivariable logistic regression analysis was performed to determine the association between lesion topography and prognosis after endovascular treatment at each time window.

**Results:**

A total of 1040 patients (mean age 68 years, 58.7% male) were included in this cohort analysis, with 564 in the early (265 with good prognosis versus 299 with poor prognosis) and 476 in the extended (218 with good prognosis versus 258 with poor prognosis) time window. For the early time window strokes, involvement of the insula (OR: 1.636, 95% CI: 1.130–2.367), caudate nucleus (OR: 1.730, 95% CI: 1.012–2.957), and the M6 (OR: 1.773, 95% CI: 1.041–3.022) were independent factors associated with poor prognosis at 90 days after adjusting for age, hypertension, diabetes, baseline NIHSS, and the time from onset to puncture. In the extended window, the M3 (OR: 2.153, 95% CI: 1.110–4.175), and the M5 (OR: 1.775, 95% CI: 1.190–2.649) were independent prognostic factors.

**Conclusions:**

Different infarct regions have varying predictive power for poor outcomes in large vessel occlusion stroke patients with different time windows. This outcome may help to identify treatment-eligible patients who are in greatest need of rapid reperfusion treatment.

**HIGHLIGHTS**

- **Research Design**: Conducted a *post-hoc* analysis of the DEVT and RESCUE BT studies to evaluate the relationship between infarct location (assessed by ASPECTS regions) and functional outcome in large vessel occlusion stroke patients in different time window. Clinical data and 90-day mRS scores were collected for analysis.
- **Key Regional Associations**: The insula, caudate nucleus, and M6 regions in the early window (<6 hours) were independently associated with 90 days poor outcome. In addition, in an extended window (6–24 hours), involvement of M3 and M5 regions independently increased the odds for poor outcomes.
- **Optimal Predictive Combination**: In the early window, the combination of lesion topography (insula, caudate nucleus, and M6 regions) as well as the traditional prognostic factors (age, diabetes, and baseline NIHSS score) had the highest area under the curve (AUC = 0.757). In the extended window, the model that combined M3 and M5 regions and traditional prognostic factors (age, hypertension, and baseline NIHSS score) had an AUC of 0.737.

## Background

Large vessel occlusion (LVO) is responsible for 30% of acute ischemic strokes and is associated with high levels of stroke-related mortality and disability[1]. The advent of endovascular thrombectomy (EVT) has revolutionized the field of ischemic stroke treatment in both early and late time windows. Since 2015, randomized trials have thus far confirmed EVT as the standard care for anterior large artery occlusion^[2–6]^. Nevertheless, even with high rates of recanalization, almost half of the patients fail to achieve functional independence^[7,8]^. Understanding the prognostic factors, therefore, will help clinicians identify the high-risk population and improve the therapeutic effect and clinical outcomes of EVT.

The Alberta Stroke Program Early CT Score (ASPECTS) is a standardized semi-quantitative CT grading system used to assess the structural infarct size and location, and has been shown to be a strong prognostic factor in patients with anterior circulation LVO^[9,10]^. Specifically, stroke patients with a low pre-treatment ASPECTS are more likely to have a poor outcome. However, such an approach ignores the heterogeneity of different lesion topography (i.e., location of infarcted tissue). Some areas are more eloquent and thus more clinically relevant to functional outcome than others due to their functional anatomy, e.g., multiple cortical and subcortical areas in motor function[11]. The ASPECTS treats all 10 areas equally; therefore, patients with the same composite ASPECTS may present different lesion patterns and various functional consequences. Indeed, it has been shown that the variation of prognosis over lesion area based on the ASPECTS regions from baseline NCCT, though with inconsistent findings^[12–15]^.

The aim of this study was to determine which regions captured by CT-ASPECTS before EVT were related to 90-day poor outcome and whether there was a difference in these critical regions according to the different time windows.

To this purpose, we merged two large acute anterior circulation stroke datasets (DEVT trial and RESCUE BT trial) of patients treated with endovascular thrombectomy (EVT) for large vessel occlusion and who had a CT before EVT. We hypothesized that the critical regions associated with 90-day poor outcomes would differ between the early and extended window. There were no uses of artificial intelligence throughout the whole manuscript, and it complies with the TITAN 2025 guidelines[16].

## Methods

### Ethics

The current study was approved by the local ethics committee. Written informed consent was obtained from all participants or their relatives before enrollment in the two trials. This clinical trial adheres to the applicable CONSORT guidelines[17]. The trial data are available from the corresponding author upon reasonable request.

### Study design

This was a *post-hoc* analysis of data collected from the DEVT Randomized Clinical Trial (Chinese Clinical Trial Registry Identifier: ChiCTR-IOR-17013568) and the RESCUE BT study (Chinese Clinical Trial Registry Identifier: ChiCTR-INR-17014167). The DEVT Randomized Clinical Trial was a multicenter, randomized noninferiority trial in patients with ischemic stroke treated with intravenous thrombolysis plus EVT or EVT alone. The RESCUE BT study was a multicenter randomized controlled study evaluating the effect of EVT combined with tirofiban versus placebo in ischemic stroke patients. The inclusion criteria of DEVT were as follows: patients >18 years old who arrived at the hospital within 4.5 hours after the onset of stroke, the occluded artery was the intracranial internal carotid artery or the M1 segment of the middle cerebral artery. The inclusion criteria of RESCUE BT were as follows: patients >18 years old who arrived at the hospital within 24 h after the onset of stroke, the baseline NIHSS score ≤ 30, and the baseline ASPECTS score ≥ 6, the occluded artery is the intracranial segment of the internal carotid artery and/or the M1/M2 segment of the middle cerebral artery.

Only patients who had infarcts in at least one ASPECTS region (ASPECTS ≤ 9) were included in this *post-hoc* analysis. Based on the time from last known well to arterial puncture, patients were dichotomized into early window (0–6 hours) and extended window (6–24 hours).

### Image analysis

All imaging data were assessed by an independent imaging core lab. The Fast-Processing of Ischemic Stroke software (version 1.0.22), an automated image postprocessing system approved by the Chinese National Medical Products Administration, was used to evaluate the baseline ASPECTS on CT imaging.

### Outcome measures

The functional outcome was assessed with the use of the modified Rankin Scale (mRS) at 90 days. Adjudication was based on the central evaluation by two mRS-certified neurologists who were blinded to treatment randomization and who reviewed the video or voice recordings elicited using a structured assessment. If video or voice recordings were unavailable, outcomes were determined in person by the local investigators, who were also unaware of the treatment assignments.

The mRS scores at 90 days were dichotomized into favorable and poor outcomes. Poor outcome was defined as an mRS score of 3–6 points according to previous definitions.

### Statistical analysis

All statistical analyses were performed with SPSS software version 23.0 (IBM Corp., Armonk, NY) and R software (version 1.3.1093). Continuous variables are expressed as mean (SD) values or median (interquartile range [IQR]) values, as appropriate, and categorical variables are expressed as counts (percentages). Statistical significance was determined by the χ^2^ test, the Fisher exact test, the *t* test, the *Z* test, and the Mann–Whitney test, as appropriate. Multivariate logistic regression models were built to identify independent predictors involving parameters with *P* < 0.1 in the univariate regression analysis. We constructed different comprehensive models by combining the ASPECTS-region characteristics and traditional clinical risk factors based on the entire population and patients with various time windows. The net benefit of the model at particular thresholds will also be examined using decision curve analysis and compared to treat-all and treat-none decisions. All *P* values were two-tailed, with statistical significance defined as *P* < 0.05.

## Results

### Baseline characteristics

The pooled cohort consisted of 1040 patients with available CT before EVT and F-ASPECTS ≤ 9, including 204 patients from the DEVT trial and 836 from the RESCUE BT trial (Supplemental Digital Content Table S1, available at: http://links.lww.com/JS9/F205). Of the 1040 patients, 58.7% (610 individuals) were male, with a median age of 68 years (IQR, 58–75 years), and the median baseline F-ASPECTS of 7 (IQR 6–8). There were 564 patients (54.2%) in the early window group, and 476 patients (45.8%) in the extended window group. Baseline clinical characteristics as well as ASPECTS region involvement are summarized in Table 1.

**Table 1:** Table of characteristics according to time from stroke onset or last known well to endovascular treatment among patients selected

Variable	Entire, *n* (%)	Early window, n (%)	Extended window, n (%)	*P* value
	Median (IQR) or mean ± SD			
		Median (IQR) or mean ± SD	Median (IQR) or mean ± SD	
	(*n* = 1040)	(*n* = 564)	(*n* = 476)	
Baseline characteristics				
Age (yr), median (IQR)	68 (58–75)	70 (60–76)	66 (56–74)	<0.001
Sex, *n* (%)				<0.001
Female	430 (41.3)	261 (46.3)	169 (35.5)	
Male	610 (58.7)	303 (53.7)	307 (64.5)	
Smoking current/past	241 (23.2)	107 (19.0)	134 (28.2)	<0.001
Hypertension	590 (56.7)	334 (59.2)	256 (53.8)	0.078
Hyperlipidemia	147 (14.1)	79 (14.0)	68 (14.3)	0.898
Diabetes mellitus	217 (20.9)	122 (21.6)	95 (20.0)	0.508
Atrial fibrillation	487 (37.2)	269 (47.7)	118 (24.8)	<0.001
Procedural and clinical results, *n* (%)				
Baseline NIHSS score, median (IQR)	16 (12–20)	16 (13–20)	15 (11–19)	<0.001
ICA intracranial	211 (20.3)	130 (23.0)	81 (17.0)	0.016
MCA-M1	695 (66.8)	374 (66.3)	321 (67.4)	0.701
MCA-M2	134 (12.9)	60 (10.6)	74 (15.5)	0.019
Onset to puncture	325 (215–560)	225 (170–280)	587 (460–785)	< 0.001
Puncture to recanalization	69 (42–108)	65 (40–104)	70 (45–116)	0.065
eTICI 2b50-3	937 (90.3)	508 (90.1)	429 (90.1)	0.976
ASPECTS regions				
M1 region	159 (15.3)	89 (15.8)	70 (14.7)	0.632
M2 region	408 (39.2)	240 (42.6)	168 (35.3)	0.017
M3 region	162 (15.6)	104 (18.4)	58 (12.2)	0.006
M4 region	308 (29.6)	168 (29.8)	140 (29.4)	0.895
M5 region	504 (48.5)	283 (50.2)	221 (46.4)	0.228
M6 region	144 (13.8)	84 (14.9)	60 (12.6)	0.287
Caudate nucleus	165 (14.0)	82 (14.5)	83 (17.4)	0.203
Internal capsule	447 (43.0)	234 (41.5)	213 (44.7)	0.290
Lentiform nucleus	317 (30.5)	141 (25.0)	176 (37.0)	< 0.001
Insula	567 (54.5)	306 (54.3)	261 (54.8)	0.852
ASPECTS, median (IQR)	7 (6–8)	7 (6–8)	7 (6–8)	0.673
Deep	137 (13.2)	73 (12.9)	64 (13.4)	0.811
Cortical	386 (37.1)	225 (39.9)	161 (33.8)	0.044
Deep + cortical	517 (49.7)	266 (47.2)	251 (52.7)	0.074
3-months outcome				
mRS	3 (1–4)	3 (1–4)	3 (1–4)	0.679
mRS 3–6	557 (53.6)	299 (54.0)	258 (54.2)	0.702
Mortality	191 (18.4)	110 (19.5)	81 (17.0)	0.302
sICH	86 (8.3)	46 (8.2)	40 (8.4)	0.885

sICH, Symptomatic intracerebral hemorrhage; deep, Caudate nucleus, Internal capsule and Lentiform nucleus; cortical, M1 region, M2 region, M3 region, M4 region, M5 region, M6 region and Insula; ICA, Internal Carotid Artery; MCA, Middle Cerebral Artery; eTICI, expanded Thrombolysis in Cerebral infarction.

Compared with patients with good outcomes, those with poor prognosis were older (early: 73 [65–78] versus 66 [57–74], *P* < 0.001; extended: 69 [61–77] versus 63 [53–71], *P* < 0.001), had more hypertension (early: 191 [63.9%] versus 143 [54.0%], *P* = 0.017; extended: 158 [61.2%] versus 98 [45.0%], *P* < 0.001), diabetes (early: 80 [26.8%] versus 42 [15.8%], *P* = 0.002; extended: 61 [23.6%] versus 34 [15.6%], *P* < 0.029), and higher NIHSS scores (early: 18 [15–21] versus 15 [11–18], *P* < 0.001; extended: 17 [13–20] versus 13 [9–17], *P* < 0.001) both in the early and extended windows. In the extended window, patients with good outcomes had more smoking (71 [32.6%] versus 63 [24.4%], *P* = 0.049) and less atrial fibrillation (44 [20.2%] versus 74 [28.7%], *P* = 0.032), but not in the early window (*P* = 0.362) (Supplemental Digital content, eTable 2, Available at: http://links.lww.com/JS9/F205).

### Stroke regions according to the ASPECTS

Among the patients, 517 (49.7%) had stroke lesions involving both the cortex and deep regions, 137 (13.2%) had pure deep lesions, and 386 (37.1%) had pure cortex lesions (Table 1).

The lentiform nucleus, a deep-seated structure, was more frequently affected in the extended window compared to the early window (176 [37.0%] versus 141 [25.0%], *P* < 0.001). Conversely, the M2 (240 [42.6%] versus 168 [35.3%], *P* = 0.017) and M3 (104 [18.4%] versus 58 [12.2%], *P* = 0.006) regions were more commonly involved in the early window than in the extended window. No significant differences were detected in other comparisons.

### Identification of regions related to the 3-month outcome

As a first step, we performed univariate logistic regression analyses to explore the clinical characteristics associated with poor outcomes at 3 months. Advanced age, history of hypertension, history of diabetes, and baseline NIHSS score were associated with poor outcomes in both the early and extended time windows. Furthermore, women and atrial fibrillation were also associated with poor prognosis in the extended time window (Table 2).Afterward, we performed univariate logistic regression analyses to determine the weight of each region separately for the 3-month poor outcome. In the early window, the M1, M2, M6, and Insula regions were found to be significant, with the M6 having the highest OR for explaining poor outcomes. In the extended window, the M2, M3, M4, M5, and M6 were significant, and the highest OR to explain poor outcome was attributed to the M3 regions. (Table 3).

**Table 2:** Univariate logistic regression analysis of risk factors for poor prognosis (mRS = 3–6) at 3 months

	Early window (*n* = 564)			Extended window (*n* = 476)		
Variables	OR	95% CI	*P* value	OR	95% CI	*P* value
Age	1.049	1.032–1.066	<0.001	1.046	1.029–1.063	<0.001
Men	0.803	0.575–1.120	0.197	0.540	0.367–0.793	0.002
Atrial fibrillation	1.166	0.837–1.625	0.363	1.590	1.038–2.437	0.033
Hypertension	1.509	1.076–2.115	0.017	1.935	1.342–2.790	<0.001
Hyperlipidemia	1.134	0.703–1.829	0.607	1.082	0.646–1.815	0.764
Diabetes	1.940	1.278–2.944	0.002	1.676	1.052–2.688	0.030
Baseline NIHSS	1.158	1.114–1.204	<0.001	1.123	1.081–1.167	<0.001

**Table 3:** Univariate logistic regression analysis of each region for poor prognosis (mRS = 3–6) at 3 months

	Early window (*n* = 564)			Extended window (*n* = 476)		
Regions	OR	95% CI	*P* value	OR	95% CI	*P* value
M1	1.715	1.073–2.741	0.024	1.415	0.842–2.378	0.19
M2	1.736	1.237–2.436	0.001	1.896	1.2281–2.791	0.001
M3	1.458	0.945–2.250	0.088	2.707	1.459–5.023	0.002
M4	1.268	0.881–1.823	0.201	1.584	1.059–2.370	0.025
M5	1.12	0.804–1.559	0.503	1.806	1.252–2.605	0.002
M6	2.076	1.270–3.395	0.004	1.817	1.027–3.213	0.040
I	1.669	1.195–2.331	0.003	0.801	0.557–1.152	0.232
L	1.222	0.832–1.793	0.307	1.183	0.813–1.720	0.380
IC	0.97	0.693–1.356	0.857	0.699	0.486–1.005	0.054
C	1.552	0.960–2.511	0.073	1.126	0.699–1.815	0.626

We conducted tests of collinearity among these regions, and collinearity was not significant. Clinical characteristics and regions with a *P* value < 0.1 in the univariate analysis were enrolled in the multivariable logistic regression (forward stepwise method). For the early time window strokes, involvement of the insula (OR: 1.636, 95% CI: 1.130–2.367), caudate nucleus (OR: 1.730, 95% CI: 1.012–2.957), and the M6 (OR: 1.773, 95% CI: 1.041–3.022) were independent factors associated with the poor prognosis at 90 days after adjusting for age, hypertension, diabetes, baseline NIHSS, and the time from onset to puncture. In the extended window, the M3 (OR: 2.153, 95% CI: 1.110–4.175), and the M5 (OR: 1.775, 95% CI: 1.190–2.649) were independent prognostic factors (Table 4).

**Table 4:** Multivariate logistic regression analysis for poor prognosis (mRS = 3–6) at 3 months

	Early window (*n* = 564)			Extended window (*n* = 476)		
Variables	OR	95% CI	*P* value	OR	95% CI	*P* value
Age	1.042	1.025–1.060	<0.001	1.033	1.016–1.051	<0.001
Hypertension				1.934	1.294–2.890	0.001
Diabetes mellitus	2.024	1.279–3.204	0.003			
Baseline NIHSS	1.140	1.095–1.187	<0.001	1.113	1.069–1.160	<0.001
M3				2.153	1.110–4.175	0.023
M5				1.775	1.190–2.649	0.005
M6	1.773	1.041–3.022	0.035			
Insula	1.636	1.130–2.367	0.009			
Caudate nucleus	1.730	1.012–2.957	0.045			

**Figure 1.**
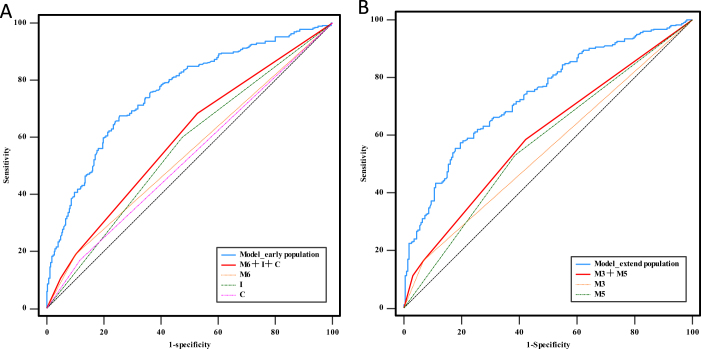
Receiver operating characteristic curves of the comprehensive model and ASPECTS regions for predicting poor prognosis at 3-month. (A) ROC curves of the early time window. (B) ROC curves of the extended time window.

In the early window, the AUC values for the Insula, caudate nucleus, and M6 regions were 0.563 (95% CI: 0.521–0.605), 0.527 (95% CI: 0.485–0.569), and 0.544 (95% CI: 0.502–0.586), respectively. The combination of lesion topography (insula, caudate nucleus, and M6 regions) as well as the traditional prognostic factors (age, diabetes, and baseline NIHSS score) had the highest AUC (0.757, 95% CI: 0.720–0.792), which was significantly higher than that of insula (Z = 7.641, *P* < 0.001), caudate nucleus (Z = 9.855, *P* < 0.001), and M6 (Z = 9.903, *P* < 0.001) respectively (Fig. 1A).

In the extended window, the AUC values of the M3 and M5 regions were 0.549 (95% CI: 0.503–0.594) and 0.573 (95% CI: 0.527–0.618), respectively. The model which combined M3 and M5 regions and traditional prognostic factors (age, hypertension and baseline NIHSS score) had an AUC of 0.737 (95% CI: 0.695–0.776), which was higher than that of the M3 region (Z = 8.332, *P* < 0.001) and M5 regions (Z = 6.228, *P* < 0.001) (Fig. 1B).

In addition, we conducted sensitivity analyses adjusting for treatment heterogeneity (including the use of rt-PA and tirofiban) and reperfusion levels; the results remained consistent with our primary finding (Supplemental Digital Content Tables S3 and S4, available at: http://links.lww.com/JS9/F205).

The decision curve analysis of the comprehensive prognosis model for 3-month outcome in the early and extended windows was visualized in Supplemental Digital Content Figure S1, available at: http://links.lww.com/JS9/F205 The models that composed the lesion topography and the traditional prognostic factors yielded a greater net benefit for every probability threshold compared with the characteristics of ASPECTS, and never did worse than treating everyone (grey line) and treating no one (thin black line at net benefit = 0), again demonstrating the superiority of the model in terms of clinical relevance.

## Discussion

ASPECTS has demonstrated reliability and reproducibility in assessing patients with AIS. Furthermore, there is evidence that the total score of ASPECTS is informative in guiding decisions about whether to proceed with endovascular treatment. However, while some studies suggest that specific regions of the ASPECTS system may have utility in patient selection, that is not a universally adopted practice. In this patient-level analysis of two randomized clinical trials, we have characterized the prognostic impact of ischemia in specific regions of the ASPECTS system in patients who underwent EVT after baseline imaging. The most important finding of our study is that the infarction in the insula, caudate nucleus, and M6 ASPECTS regions predicts a poor outcome (mRS of 3–6) in patients with an early time window. Moreover, in the extended time window, M3 and M5 regions have independent effects on the 90-day clinical outcome.

Given the increasing use of EVT in the reperfusion therapy of acute ischemic stroke with LVO, it’s important to stratify the risk of poor outcome based on the lesion topography for optimal therapy. Our study showed that not all ASPECTS regions are of equal clinical importance. We found infarcts involving the insula were the most prevalent (54.5%), which aligns with the results of previous studies^[18,19]^. This can be attributed to the insula’s blood supply, which is exclusively from the deep perforating branches of the MCA, primarily the M2 segment, rather than from the pial collateral circulation of the anterior or posterior cerebral arteries^[20,21]^. The poor collateral circulation simultaneously leads to a higher ischemic vulnerability to hypoperfusion in the hyperacute phase, and is more likely to progress into surrounding penumbral tissue[22]. Indeed, our data suggest that patients with insular involvement appear to experience more severe and extensive strokes with higher NIHSS scores and lower ASPECTS (Supplemental Digital Content Table S5, available at: http://links.lww.com/JS9/F205). Thus, it is not too surprising to find that the involvement of the insula was associated with the poor prognosis at 90 days in patients with an early treatment window. Furthermore, the insula plays a central role in neural network connectivity and is typically involved in autonomic regulation, sensory processing, and cognitive control, which was supported by mathematical modeling of its structural and functional organization[23]. Therefore, insular lesions might induce autonomic imbalance and subsequently cardiac arrhythmia, like atrial fibrillation, other ECG abnormalities, and even Takotsubo-like cardiomyopathy due to the dysregulation of vagal cardiac parasympathetic neuronal pool[24], which may also contribute to their poor outcomes.

For the caudate nucleus, its role in the 3-month prognosis of patients in the early treatment window is somewhat surprising. Two explanations for this phenomenon can be proposed. The primary focus is on its structural connectivity and its functional role. As an associative structure, the caudate nucleus has important connections with the frontal and parietal lobes and plays an essential role in motor, behavioral, and executive dysfunction[25], as well as being associated with cognitive changes[18]. Second, as part of the gray matter, the caudate nucleus is highly dependent on energy supply and has greater metabolic demands than white matter^[26,27]^, providing support for higher vulnerability to hypoperfusion in the hyperacute phase. Despite endovascular treatment and successful reperfusion, neuronal necrosis in the caudate nucleus may not be reversible[28], predicting difficulty in recovering motor and cognitive functions. Meanwhile, our study also found that the M6 region was associated with poor prognosis in patients with an early time window. The M6 region belongs to the watershed area and has fewer collateral pathways compared to other regions. Indeed, the CBF in the M6 region was significantly lower than that of the contralateral side in patients with MCA occlusion[29]. A previous study by Phan *et al*. also suggested that M6 involvement was associated with poor outcome in the elderly[13].

In the extended window, a poor outcome was observed when M3 (inferior parietal) and M5 (frontoparietal) were affected. Damage to M3 may result in neglect, which in turn disturbs the processing of external stimuli or the patient’s performance in motor rehabilitation, and these are also reflected in higher mRS scores^[25,30]^. In addition, the M5 is a frontoparietal region, which plays an important role in regulating language function and higher cognitive functions through the arcuate fasciculus, U fibers, and superior longitudinal fasciculus^[25,31–33]^. Anatomically, the blood supply to the M3 and M5 region is rich, mainly including the cortical branches of the middle cerebral artery and the network of pial collateral with the anterior cerebral artery and the posterior cerebral artery. During the 6–24 hours after stroke, the pial collateral gradually fails, leading to ischemia in M3 or M5 regions. Even with vessel recanalization, the delayed injury cannot be reversed.

According to recent clinical trials, despite the high recanalization rate of EVT, the prognosis could still be suboptimal^[34,35]^. How to screen patients who would benefit the most is a question that remained unaddressed. Previous studies suggested that the total ASPECTS correlates with functional outcome and can be used as a screening tool^[9,10]^. We present evidence that the prognosis also depends on the regions of infarction. Furthermore, different brain regions might have distinct contributions within different time windows. Our study offers a novel perspective for assessing prognosis and supplies a potential avenue for screening patients who may benefit from the EVT.

However, this study has several limitations. First, this was a retrospective analysis based on the data from 2 randomized trials, and selection bias was inevitable. Second, the analysis was carried out on the ischemic stroke of the anterior circulation. Therefore, the results of this study are not applicable to patients with posterior circulation stroke. Third, given the limited number of patients with ASPECTS less than 6, these results should be interpreted with caution for patients with large core infarction.

## Conclusions

Not all infarct locations have the same weight in predicting stroke prognosis at 90 days in patients with anterior circulation LVO. Moreover, these regions depend on the time window. The involvement of the insula, caudate nucleus, and M6 regions in the early window (0–6 hours) was independently associated with poor outcome at 3 months. In addition, in the extended window (6–24 hours), involvement of M3 and M5 regions independently increases the odds for poor outcomes. Our study highlights the important role of these regions and the time window in brain function and processes leading to disability.

## Data Availability

The trial data are available from the corresponding author upon reasonable request.

## Research registration unique identifying number (UIN)

Chinese Clinical Trial Registry Identifier: ChiCTR-IOR-17013568 and Chinese Clinical Trial Registry Identifier: ChiCTR-INR-17014167.
